# Risk factors of colorectal cancer in middle-aged and elder adults in China: findings from the China health and retirement longitudinal study

**DOI:** 10.3389/fmolb.2025.1333834

**Published:** 2025-04-25

**Authors:** Lin Zhang, Yuyan Zhang, Yujia Huo, Yang Zhao, Aimin Xu, Zhining Liu, Qiaojun Hong, Huiming Tu, Junjie Huang, Li Liu

**Affiliations:** ^1^ The School of Public Health and Preventive Medicine, Monash University, Suzhou, China; ^2^ Suzhou Industrial Park Monash Research Institute of Science and Technology, Monash University, Suzhou, China; ^3^ Monash University-Southeast University Joint Research Institute (Suzhou), Southeast University, Nanjing, China; ^4^ The George Institute for Global Health, University of New South Wales, Sydney, NSW, Australia; ^5^ Department of Statistics, College of Big Data and Software Engineering, Zhejiang Wanli University, Ningbo, China; ^6^ Anhui Medical University Affiliated Hospital, Hefei, China; ^7^ Department of Oncology, Anhui No. 2 Provincial People’s Hospital, Hefei, China; ^8^ Department of Gastroenterology, Affiliated Hospital of Jiangnan University, Wuxi, China; ^9^ The Jockey Club School of Public Health and Primary Care, Faculty of Medicine, Chinese University of Hong Kong, Shatin, China; ^10^ Big Data Center, Affiliated Hospital of Jiangnan University, Wuxi, China

**Keywords:** middle-aged and elder adult, Chinese population, risk factor, CHARLS, colorectal caner

## Abstract

**Objective:**

This study aims to identify risk factors of colorectal cancer in a middle-aged and elder Chinese population over 45 years old and to provide evidence for preventing colorectal cancer in China.

**Method:**

The China Health and Retirement Longitudinal Study (CHARLS) is a nationally representative cohort used for research on demography, lifestyle and characteristics of colorectal cancer population. The logistic regression model was used to estimate the odds ratio (OR) and corresponding confidence interval (95% CI) using the maximum likelihood method. Univariate logistic regression was performed with the ORs of each risk factor and its association with incidence of colorectal cancer. Risk factors significant in univariate logistic regression were further evaluated by multivariate logistic regression. Cox proportional hazards model estimated the hazard ratio (HR) of each risk factor and its association with incidence of colorectal cancer.

**Results:**

In the univariable analysis, sex (OR = 2.31, 95% CI: 1.00–5.36, p = 0.05), smoking (OR = 2.30, 95% CI: 1.03–5.13, p = 0.04), age of quit drinking (OR = 1.07, 95% CI: 1.01–1.14, p = 0.02) and chronic lung disease (OR = 2.79, 95% CI: 1.11–6.99, p = 0.03) were associated with colorectal cancer which was also included in the multivariable analysis. However, probably because of the small sample size of colorectal cancer patients, no indicator was confirmed to be risk factor of colorectal cancer in the multivariable logistic regression. The univariate analysis of the Cox model indicated that smoking (HR = 2.30, 95%: 1.03–5.13, p = 0.04) and chronic lung disease (HR = 2.79, 95%: 1.11–6.97, p = 0.03) were associated with incidence of colorectal cancer. Similar to the results of multiple linear regression, no indicator was confirmed to be risk factors of incidence of colorectal cancer in the multivariable Cox model.

**Conclusion:**

In the univariate analysis, we identified significant associations between colorectal cancer and factors such as smoking and chronic lung disease. However, these associations did not hold in the multivariate analysis due to limitations in sample size. This suggests the need for further validation of these potential risk factors in larger-scale studies.

## Introduction

Colorectal cancer (CRC) is a significant global health issue, with increasing incidence and mortality rates worldwide (Liu et al., 2023). According to the results of the World Health Organization International Agency for Research on Cancer (IARC) 2020 report, among the new cancer cases in the world in 2020, the number of new cases of CRC ranked third, and the mortality accounted for 9.4% of cancer deaths (Collaborators, 2020). CRC contributes significantly to impaired quality of life and loss of independence among middle-aged and elder individuals (Liu et al., 2023). The burden of CRC is particularly prominent in China, the largest developing country in the world, where the prevalence of CRC is substantial (Wang et al., 2020). Based on the statistical data provided by the IARC, the incidence of CRC in China has escalated to 555,477 new cases in 2020, ranking second in the number of new cancer cases in the country. Furthermore, the mortality rate due to CRC has been reported to be 283,751, accounting for 9.5% of cancer-related deaths in China (Collaborators, 2020). CRC among middle-aged and elder individuals in China is particularly important, as this population is disproportionately affected by the disease (Wang et al., 2020). The risk of CRC increases significantly with age, especially after the age of 45 years (Ma et al., 2023). As the population in China continues to age, the prevalence of CRC is expected to grow, adding to the already significant disease burden (Li et al., 2022).

Despite the significant burden of CRC in China, limited studies have estimated its risk factors, especially among the middle-aged and elder population (Wang et al., 2020). Previous studies, such as the Study on Global Ageing and Adult Health, have provided some insights into the prevalence of CRC but were not specifically designed for middle-aged and older adults and were conducted in only a limited number of provinces (Wang et al., 2020). Therefore, there is a need for a comprehensive study to estimate the risk factors and CRC among middle-aged and older Chinese adults using national representative data (Li et al., 2022).

The China Health and Retirement Longitudinal Study (CHARLS) is uniquely suited for studying CRC risk factors due to several key strengths. First, CHARLS has a large sample size, with over 17,708 participants aged 45 and above, ensuring that the study findings are representative of the broader Chinese population (Zhao et al., 2014). Second, the study collects comprehensive data on various aspects of health, lifestyle, and socioeconomic status, providing a rich dataset for identifying potential risk factors (Chen X. et al., 2019). Third, the longitudinal design of CHARLS allows for the assessment of long-term risk factors and their impact on CRC incidence over time, which is crucial for understanding the etiology of the disease (Wang et al., 2020). These strengths make CHARLS an ideal data source for our study, enabling us to conduct a thorough and robust analysis of CRC risk factors in middle-aged and older Chinese adults.

Lifestyle and cultural practices in China, such as diet, physical activity, and smoking habits, play a significant role in CRC risk and may differ from global trends. Traditional Chinese diets often include high consumption of processed foods and low intake of dietary fiber, which are known risk factors for CRC (Liu et al., 2023). Physical activity levels in China are generally lower compared to Western countries, with a significant portion of the population leading sedentary lifestyles (Chen et al., 2022). Additionally, smoking rates in China are among the highest globally, with a significant impact on health outcomes, including CRC (Jiang et al., 2024; Xu et al., 2024). These lifestyle and cultural practices are particularly relevant to the Chinese context and highlight the need for targeted interventions to address these risk factors. The role of environmental and genetic factors in driving cancer progression has been highlighted in prior reviews, emphasizing the complexity of carcinogenesis pathways, as reviewed comprehensively (Bhat et al., 2024).

Identifying risk factors associated with CRC is crucial for developing targeted preventive strategies. Age, gender, obesity, smoking, and physical activity have been identified as potential risk factors for CRC (Li et al., 2022). However, most of these studies have focused on populations outside of China, highlighting the need for specific research in the Chinese context (Li et al., 2022). In light of these gaps in knowledge, the current study aims to identify risk factors associated with CRC among middle-aged and older Chinese adults. This study were conducted by using baseline data from the China Health and Retirement Longitudinal Study (CHARLS) and the longitudinal data of the CHARLS with both prevalence and incidence risk of colorectal cancer (Li et al., 2022).

To provide a clearer understanding of the research direction, we have formulated specific hypotheses based on existing literature and the unique aspects of our study population. We hypothesize that: Smoking and alcohol consumption are significant risk factors for CRC in middle-aged and older Chinese adults; Obesity, as measured by body mass index (BMI) and waist circumference, is associated with an increased risk of CRC; Physical inactivity and sedentary lifestyle contribute to the incidence of CRC; Dietary factors, such as high consumption of processed foods and low intake of dietary fiber, are linked to higher CRC risk. These hypotheses will guide our analysis and help identify potential areas for targeted interventions.

Our study aims to fill the following specific voids in the current literature: First, the lack of longitudinal data on CRC risk factors among middle-aged and older adults in China. Most existing studies are cross-sectional or have limited follow-up periods, which do not allow for the assessment of long-term risk factors. Second, the underrepresentation of this demographic in previous studies. Many studies focus on younger populations or do not specifically target middle-aged and older adults, who are at higher risk for CRC. Third, limitations in the methodologies used in prior research. Some studies lack comprehensive data collection or fail to account for potential confounding variables. To overcome these limitations, our study utilizes the CHARLS, a nationally representative longitudinal cohort, to provide a robust analysis of CRC risk factors over time. This approach allows us to identify potential risk factors and their long-term impact on CRC incidence in the Chinese population.

In addition to these gaps, it is crucial to consider region-specific factors that may uniquely influence CRC risk in China. For instance, lifestyle factors such as high consumption of processed foods and low intake of dietary fiber are prevalent in certain regions, which may contribute to the higher incidence of CRC (Liu et al., 2023). Environmental factors, including high levels of air pollution, have also been linked to increased cancer risk (Jiang et al., 2024; Xu et al., 2024). Furthermore, genetic predispositions and variations in the Chinese population may play a role in CRC susceptibility (Wang et al., 2020). These unique factors highlight the need for research that specifically addresses the Chinese context, as global trends may not be directly applicable.

By addressing these research objectives, this study enhances our understanding of CRC among middle-aged and older individuals in China. The findings have significant implications for public health initiatives, resource allocation, and the development of targeted preventive strategies. Specifically, identifying region-specific risk factors and leveraging longitudinal data can inform the creation of tailored interventions, such as smoking cessation programs, dietary interventions, and increased physical activity initiatives. These measures can be particularly effective for middle-aged and older adults, who are at higher risk for CRC. Additionally, our findings can guide healthcare planning and resource allocation, ensuring that preventive measures and screening programs are effectively implemented to reduce the burden of CRC in China.

## Method

### Study population and design

The China Health and Retirement Longitudinal Study (CHARLS) is a nationally representative survey conducted in China aimed at understanding the health and economic wellbeing of the Chinese population aged 45 and above (Zhao et al., 2014). The survey design of CHARLS is comprehensive and incorporates multiple aspects, including demographics, health status, lifestyle behaviors, economic circumstances, and social networks to provide a holistic understanding of the participants’ lives (Zhao et al., 2014).

The survey follows a multistage, stratified, and cluster sampling design to ensure the representativeness of the population (Chen X. et al., 2019). The baseline survey was conducted in 2011, covering 17 provinces and municipalities in China, and included over 10,000 households and 17,708 individuals (Zhao et al., 2014). The sample was selected using a three-stage process, starting with selecting counties or districts, followed by townships or streets, and finally, households within the selected areas (Zhao et al., 2014). Within each household, all eligible individuals aged 45 and above were invited to participate. Follow-up surveys were conducted in 2013, 2015, and 2018 to collect updated information on the participants’ health status and any occurrences of colorectal cancer. Out of the initial sample size of 17,708 participants, 21 individuals who had colorectal cancer prior to 2011 were excluded from the study. Consequently, the final number of exposed individuals, those with colorectal cancer was 25, while the remaining 17,662 individuals constituted the unexposed group.

The survey instrument for CHARLS consists of a household questionnaire and individual questionnaires (Zhao et al., 2014). The household questionnaire collects information on household composition, assets, income, and expenditures. Meanwhile, the individual questionnaire covers various topics, including demographic characteristics, health status, healthcare utilization, lifestyle behaviors, and social networks. The survey utilizes computer-assisted personal interviewing (CAPI) techniques, allowing for efficient and accurate data collection (Zhao et al., 2014).

### Inclusion and exclusion criteria

Participants included in this study were individuals aged 45 and above who participated in the CHARLS. We excluded individuals who had a diagnosis of CRC before 2011 to ensure that our analysis focused on incident cases and to avoid potential biases related to survivorship or treatment effects. No additional exclusions were made based on comorbid conditions or treatment history, ensuring a broad representation of the population.

### Risk factors of colorectal cancer

The variable selection process was finalized based on extensive literature research. Several studies were reviewed to identify and evaluate relevant variables for the study. Key variables were identified based on their theoretical relevance and empirical support from previous studies. Finally, the selected variables can be categorized into two types: continuous variables and categorical variables. Continuous variables included height, weight, waist, age of quitting smoking, age of beginning smoking, triglycerides (mg/dL), HDL cholesterol (mg/dL), LDL cholesterol (mg/dL), hsCRP (mg/L). Category variables included age, sex, BMI, smoking, drinking, self-reported health status, days do vigorous/moderate/walking activities at least 10 min continuously weekly, hypertension, heart disease, stroke, dyslipidemia, diabetes, glucose≥126.0 mg/dL, HbA1c ≥ 6.5%, average hours for night sleeping, Inflammatory diseases (arthritis, asthma), other non-neoplastic diseases (chronic lung disease, stomach disease, liver disease).

Except for variables obtained through physical examination and blood sample collection analysis, all other variables were obtained through self-reported survey. Respondents were defined as having doctor-diagnosed CRC if they answered Colon or Rectum to the question, Organ or Body Part have Cancer. People were asked if they have any health problems such as high blood pressure, high cholesterol, diabetes, cancer (not including minor skin cancers), lung problems, liver problems, heart problems, stroke, kidney problems, stomach or other digestive problems (not including tumors or cancer), mental health problems, memory problems, and asthma. People who said yes to these questions were diagnosed by a doctor with these conditions. Certain continuous variables will be converted or categorized as categorical during the analysis process. Age was categorized into three groups, ≤59, 60–69 and ≥70. To ensure clarity and consistency, we defined cutoffs for categorized variables based on established guidelines and recommendations. For BMI, we used the World Health Organization (WHO) classifications: underweight (<18.5), normal (18.5–24.9), overweight (25–29.9), and obese (≥30). For sleep duration, we followed the National Sleep Foundation’s recommendations, categorizing it as short (<7 h), recommended (7–9 h), and long (>9 h). Biomarkers such as triglycerides and HDL cholesterol were measured using standard laboratory techniques, with normal levels defined as less than 150 mg/dL for triglycerides and 40 mg/dL or above for HDL cholesterol in men, and 50 mg/dL or above in women, based on the ATP III Guidelines (Expert Panel on detection et al., 2002). The questionnaire determines the classification criteria of another categorical variable.

### Definitions and diagnostic criteria

To ensure clarity and reduce ambiguity, we defined key health conditions based on standard clinical criteria and self-reported diagnoses by healthcare professionals. Chronic lung disease includes conditions such as COPD and asthma. Hypertension is defined by blood pressure readings or self-reported diagnosis. Diabetes is identified by blood glucose levels or self-reported diagnosis. Dyslipidemia is determined by cholesterol levels or self-reported diagnosis. Heart disease encompasses conditions like coronary heart disease and myocardial infarction. Stroke and inflammatory diseases like arthritis and asthma are also based on self-reported diagnoses. These definitions help maintain consistency in our analysis.

### Statistical analysis

Overall and gender-specific prevalence of CRC in each survey wave was obtained by statistical analysis procedures performed. Additionally, the overall and gender-specific prevalence of CRC was estimated. Logistic regression and Cox proportional hazards models were chosen based on the nature of the data and the study objectives. Logistic regression was used to estimate the odds ratios (OR) and corresponding 95% confidence intervals (CI) for the association between risk factors and the prevalence of colorectal cancer (CRC). This model is appropriate for binary outcomes and allows for the assessment of multiple risk factors simultaneously. The Cox proportional hazards model was employed to investigate the time-to-event data, specifically the incidence of CRC, and to estimate hazard ratios (HR) while accounting for varying follow-up times among participants. This model is particularly suited for survival analysis and provides insights into the temporal relationships between risk factors and CRC incidence.

In this study, logistic regression analysis was employed to estimate the odds ratio (OR) and corresponding 95% confidence interval (CI) using the maximum likelihood method implemented in STATA 17MP software. Univariate logistic regression was conducted to determine the ORs of each risk factor and their association with the prevalence of CRC. Subsequently, significant risk factors identified from the univariate analysis were further evaluated using multivariate logistic regression. Statistical significance was defined as p < 0.05, indicating a level of significance at which the null hypothesis can be rejected. Cox proportional hazards model was employed to investigate the factors influencing the onset time for colorectal cancer. The Cox model is a statistical technique widely used in survival analysis to investigate the relationship between predictor variables and the time-to-event outcome. It allows for the examination of the impact of multiple risk factors simultaneously while accounting for differing follow-up times among individuals. In the analysis results of the Cox model, HR is used to measure the impact intensity of different variables on the onset time of colorectal cancer, Standard error measures the uncertainty of the estimation results, and p value < 0.05 is used to judge the significance of the estimation results.

To ensure the validity of our analyses, we addressed missing data through a combination of exclusion and imputation methods. Specifically, for variables with less than 5% missing data, we used mean imputation for continuous variables and mode imputation for categorical variables. For variables with more than 5% missing data, we excluded those variables from the analysis to avoid introducing significant bias. This approach balanced the need to retain as much data as possible while minimizing the potential impact of missing data on our results.

To test the robustness of our findings, we conducted several sensitivity analyses. These included excluding outliers for continuous variables such as BMI and cholesterol levels, varying inclusion criteria for age groups and health conditions, and cross-checking self-reported data with available medical records. These analyses confirmed the robustness of our results, providing confidence in the validity of our findings.

### Ethical considerations

Ethical approval was not required for this study because it utilized de-identified, publicly available data from the CHARLS. The data used in this study were anonymized, and no personally identifiable information was accessed or used. Participant consent was obtained by CHARLS during data collection, and all participants provided informed consent for their data to be used in research. Data confidentiality was maintained through strict adherence to data privacy protocols, ensuring that all data were handled securely and in compliance with relevant regulations.

## Results

### Descriptive analysis

The proportion of people with CRC in different age groups was 52.0%, 24.0%, and 24.0% for age groups ≤59, >60 but <69 and ≥70, respectively. Among the affected population, the proportion of males was 68.0%, higher than females (32.0%). CRC patients' mean height, weight and waist are 161.4 cm, 59.9 kg and 82.6 cm, respectively. Most respondents and CRC patients were of average or obese body shape. Patients with CRC demonstrate a higher smoking prevalence, with a substantial proportion engaging in long-term smoking habits. Fewer patients with CRC have a drinking habit. Respondents generally engage in sports activities for 6–7 days per week. According to our sample’s statistical findings, individuals diagnosed with colorectal cancer did not exhibit a sleep duration exceeding 9 h per day. Basic information characteristics are shown in Table 1. CRC patients exhibit significantly elevated mean levels of total cholesterol, triglycerides, HDL cholesterol, and LDL cholesterol compared to their non-colorectal cancer counterparts. It is consistently observed that individuals diagnosed with CRC commonly present with high total cholesterol (68.4%), high triglycerides (73.7%), high HDL cholesterol (90.6%), and very high LDL cholesterol (64.9%). Biomarkers' characteristics are shown in Table 2. The respondents demonstrate a prevalent presence of asthma. Except for Hypertension, Diabetes and Arthritis, the proportion of CRC patients with other diseases is higher than that of non-CRC populations. Disease characteristics was presented in Table 3.

**TABLE 1:** Basic information characteristics of the participants by colorectal cancer status.

Variable	Category	Colorectal cancer		Total(N=17687)
		No (n=17662)	Yes (n=25)	
Baseline age group	<=59	9963 (56.6%)	13 (52.0%)	56.6%
	60–69	4729 (26.9%)	6 (24.0%)	26.9%
	>=70	2916 (16.6%)	6 (24.0%)	16.6%
Sex	Female	9199 (52.1%)	8 (32.0%)	52.1%
	Male	8450 (47.9%)	17 (68.0%)	47.9%
Height, mean (SD)		158.0 (8.9)	161.4 (8.5)	158.0 (8.9)
Weight, mean (SD)		58.8 (11.8)	59.9 (11.0)	58.8 (11.8)
Waist, mean (SD)		84.3 (12.6)	82.6 (9.6)	84.3 (12.6)
BMI	Normal (18.5–24.9)	8362 (47.7%)	13 (52.0%)	47.7%
	Low (<18.5)	946 (5.4%)	2 (8.0%)	5.4%
	Pre-obese (25–29.9)	3490 (19.9%)	3 (12.0%)	19.9%
	Obese (>=30)	4733 (27.0%)	7 (28.0%)	27.0%
Smoking	No	10668 (60.6%)	10 (40.0%)	60.5%
	Yes	6944 (39.4%)	15 (60.0%)	39.5%
Still smoke	Still	5495 (69.9%)	12 (70.6%)	69.9%
	Quit	2364 (30.1%)	5 (29.4%)	31.1%
Age of quit smoking, mean (SD)		57.7 (12.3)	59.8 (14.1)	57.7 (12.3)
Age of begin smoking, mean (SD)		22.3 (8.4)	24.5 (10.6)	22.3 (8.4)
Drinking	No	11829 (67.2%)	17 (68.0%)	67.2%
	Yes	5783 (32.8%)	8 (32.0%)	32.8%
Ever Drink	Never	11373 (75.1%)	15 (71.4%)	75.1%
	Less than once a month.	1813 (12.0%)	1 (4.8%)	12.0%
	More than once a month.	1949 (12.9%)	5 (23.8%)	12.9%
Age of quit drinking, mean (SD)		54.9 (13.9)	66.6 (8.6)	54.9 (13.9)
Age of begin drinking, mean (SD)		26.3 (12.3)	23.2 (7.0)	26.3 (12.3)
Frequency of drinking liquor	Once a month	507 (10.6%)	1 (12.5%)	10.6%
	2–3 times a month	797 (16.7%)	1 (12.5%)	16.7%
	Once a week	406 (8.5%)	0 (0.0%)	8.5%
	2–3 times a week	569 (11.9%)	0 (0.0%)	11.9%
	4–6 times a week	187 (3.9%)	0 (0.0%)	3.9%
	Once a day	1335 (27.9%)	2 (25.0%)	27.9%
	Twice a day	725 (15.2%)	3 (37.5%)	15.2%
	More than twice a day	253 (5.3%)	1 (12.5%)	5.3%
Self-comment of Health status	Excellent	10 (0.1%)	0 (0.0%)	0.1 %
	Very good	616 (3.5%)	1 (4.0%)	3.5%
	Good	1804 (10.2%)	3 (12.0%)	10.2%
	Fair	8392 (47.6%)	10 (40.0%)	47.6%
	Poor	5880 (33.4%)	9 (36.0%)	33.4%
	Very poor	914 (5.2%)	2 (8.0%)	5.2%
Days Do Vigorous Activities At Least 10 Minutes Continuously weekly	1–5 days	2640 (15.1%)	5 (20.0%)	15.1%
	6–7 Days	14816 (84.9%)	20 (80.0%)	84.9%
Days Do Moderate Activities At Least 10 Minutes Continuously weekly	1–5 days	2950 (16.9%)	7 (28.0%)	16.9%
	6–7 Days	14502 (83.1%)	18 (72.0%)	83.1%
Days Walking At Least 10 Minutes Continuously weekly	1–5 days	1815 (10.4%)	2 (8.0%)	10.4%
	6–7 Days	15710 (89.6%)	23 (92%)	89.6%
Average Hours for Night Sleeping	7–9 h	7738 (43.8%)	13 (52.0%)	43.8%
	<7 h	8732 (49.4%)	12 (48.0%)	49.4%
	>9 h	1192 (6.7%)	0 (0.0%)	6.7%

**TABLE 2:** Biomarkers characteristics of the participants by colorectal cancer status.

Variable	Category	ATP III classification (mg/dL)	Colorectal cancer	
			No (N=17662)	Yes (n=25)
Dyslipidemia			1655 (9.3%)	4 (16.0%)
Total cholesterol (mg/dL), mean (SD)			192.9 (38.9)	205.1 (50.5)
	Desirable	<200	7048 (40.0%)	6 (24.0%)
	Borderline high	200–239	3219 (18.3%)	4 (16.0%)
	High	≥240	7362 (41.8%)	15 (60.0%)
Triglycerides (mg/dL), mean (SD)			134.8 (110.3)	139.8 (79.4)
	Low	<150	8434(47.8%)	9 (36.0%)
	High	≥150	9228 (52.2%)	16 (64.0%)
HDL cholesterol (mg/dL), mean (SD)			50.9 (15.3)	51.4 (16.7)
	Low	<40	4156 (31.9%)	3 (15.0%)
	High	≥60	8852 (68.1%)	17 (85.0%)
LDL cholesterol (mg/dL), mean (SD)			116.0 (34.9)	127.5 (43.0)
	Optimal	<100	3819 (21.8%)	4 (16.7%)
	Near optimal/above optimal	100–129	4000 (22.9%)	4 (16.7%)
	Borderline high	130–159	2397 (13.7%)	1 (4.2%)
	High	160–189	851 (4.9%)	1 (4.2%)
	Very high	≥190	6414 (36.7%)	14 (58.3%)

**TABLE 3:** Other disease characteristics of the participants by colorectal cancer status.

Variable	Colorectal cancer	
	No (N=17662)	Yes (n=25)
Hypertension	4292 (24.5%)	5 (20.0%)
Heart disease	2099 (12.0%)	5 (20.0%)
Stroke	418 (2.4%)	1 (4.0%)
Diabetes	1005 (5.7%)	1 (4.0%)
Glucose≥126.0 mg/dL	7718 (43.7%)	13 (52.0%)
HbA1c≥6.5%	6587 (37.3%)	13 (52.0%)
Arthritis	11791 (67.2%)	14 (56.0%)
Asthma	16938 (96.4%)	24 (96.0%)
hsCRP (mg/L), mean (SD)	2.8 (7.6)	3.5 (3.3)
Chronic lung disease	1787 (10.2%)	6 (24.0%)
Stomach disease	3903 (22.2%)	9 (36.0%)
Liver disease	680 (3.9%)	2 (8.0%)

### Univariate logistic regression

When comparing genders, men exhibited a risk ratio of 2.31 as opposed to women, with a p-value of 0.051. Although slightly exceeding the traditional threshold for statistical significance, this result remains close to the significance level. Thus, it would be inappropriate to entirely dismiss the possibility of a significant difference in the risk ratio between men and women. No significant association was observed between age, height, weight, waist, and BMI and the incidence of colorectal cancer (Table 4).

**TABLE 4:** The association between age, gender, height, weight, waist, BMI and colorectal cancer.

Variable	Category	OR	95% CI	P value
Baseline age group	<=59	Ref		
	60–69	0.97	0.37–2.56	0.96
	>=70	1.58	0.60–4.15	0.92
Sex	Female	Ref		
	Male	2.31	1.00–5.36	0.05
Height, mean (SD)		1.05	0.95–1.10	0.09
Weight, mean (SD)		1.01	0.97–1.04	0.69
Waist, mean (SD)		0.99	0.96–1.02	0.55
BMI	Normal (18.5–24.9)	Ref		
	Low (<18.5)	1.35	0.31–6.04	0.69
	Pre-obese (25–29.9)	0.55	0.16–1.94	0.36
	Obese (>=30)	0.95	0.34–2.38	0.92

Our analysis identified significant associations between colorectal cancer (CRC) and factors such as smoking (OR = 2.30, 95% CI: 1.03–5.13, p = 0.04) and chronic lung disease (OR = 2.79, 95% CI: 1.11–6.99, p = 0.03). These findings underscore the importance of these risk factors in the development of CRC.

The likelihood of developing colorectal cancer is 2.3 times higher among smokers than non-smokers (OR = 2.30, 95% CI: 1.03–5.13, p = 0.04), indicating an increased susceptibility to colorectal cancer in smokers. However, there exists no significant correlation between smoking cessation and the incidence of colorectal cancer. The risk of developing colorectal cancer did not differ significantly between individuals who had quit smoking and those who were still smoking (OR = 0.98, 95% CI: 0.35–2.79, p = 0.98), suggesting a limited role of smoking cessation in reducing the risk of colorectal cancer (Table 5). In terms of alcohol consumption, research findings reveal no significant association between alcohol intake and the occurrence of colorectal cancer. Both individuals who never drank alcohol and regular drinkers did not exhibit a significantly elevated risk of colorectal cancer compared to non-drinkers (OR = 0.96, 95% CI: 0.42–2.23, p = 0.93). Nevertheless, a further analysis considering the frequency and age of alcohol consumption yielded intriguing results. Notably, an evident relationship was observed between the age at which alcohol consumption ceased and the incidence of colorectal cancer (OR = 1.07, 95% CI: 1.01–1.14, p = 0.02). This implies that individuals who abstain from alcohol later in life face a heightened risk of developing colorectal cancer. However, no substantial correlation was found between the age at which alcohol consumption commenced and the incidence of colorectal cancer (OR = 0.97, 95% CI: 0.92–1.03, p = 0.38).

**TABLE 5:** The association between smoking, drinking and colorectal cancer.

Variable	Category	OR	95% CI	P value
Smoking	No			
	Yes	2.30	1.03–5.13	0.04
Still smoke	Still	Ref		
	Quit	0.98	0.35–2.79	0.98
Age of quit smoking, mean (SD)		1.01	0.96–1.06	0.55
Age of begin smoking, mean (SD)		1.02	0.98–1.08	0.32
Drinking	No	Ref		
Ever Drink	Yes	0.96	0.42–2.23	0.93
	Never	Ref		
	Less than once a month.	0.42	0.06–3.17	0.40
	More than once a month.	1.95	0.71–5.36	0.20
Age of quit drinking, mean (SD)		1.07	1.01–1.14	0.02
Age of beginning drinking, mean (SD)		0.97	0.92–1.03	0.38
Frequency of drinking liquor	Once a month	Ref		
	2–3 times a month	0.64	0.04–10.20	0.75
	Once a week	—		
	2–3 times a week	—		
	4–6 times a week	—		
	Once a day	0.75	0.07–8.40	0.82
	Twice a day	2.10	0.22–20.23	0.52
	More than twice a day	2.00	0.12–32.17	0.64

The self-evaluation of health status and the duration of vigorous exercise, moderate exercise, and walking for at least 10 min, sleeping were not statistically significant for the risk of colorectal cancer (Table 6). The results of Logistic regression did not show the correlation between *Dyslipidemia, triglycerides and cholesterol* and the risk of colorectal cancer (Table 7). Chronic lung disease was significantly associated with colorectal cancer. Patients with chronic lung disease have a nearly 3-fold higher risk of developing colorectal cancer than those without lung disease (OR = 2.82, 1.28–6.25, p = 0.01). The population with other diseases did not show any changes in the risk of developing colorectal cancer (Table 8).

**TABLE 6:** The relationship between self-comment of Health status, exercise, sleeping and colorectal cancer.

Variable	Category	OR	95% CI	P value
Self-comment of Health status	Excellent	Ref		
	Very good	0.74	0.07–8.20	0.81
	Good	0.76	0.13–4.56	0.76
	Fair	0.54	0.12–2.49	0.43
	Poor	0.70	0.15–3.24	0.65
	Very poor	—		
Days Do Vigorous Activities At Least 10 Minutes Continuously weekly	1–5 days	Ref		
	6–7 Days	0.71	0.27–1.90	0.50
Days Do Moderate Activities At Least 10 Min Continuously weekly	1–5 days	Ref		
	6–7 Days	0.52	0.22–1.52	0.15
Days Walking At Least 10 Min Continuously weekly	1–5 days	Ref		
	6–7 Days	1.33	0.31–5.64	0.70
Average Hours for Night Sleeping	7–9 h	Ref		
	<7 h	0.82	0.37–1.79	0.62
	>9 h	—		

**TABLE 7:** The association between dyslipidemia, triglycerides, cholesterol and colorectal cancer.

Variable	Category	ATP III classification (mg/dL)	OR	95% CI	P value
Dyslipidemia			1.87	0.64–5.44	0.25
	Desirable	<200	Ref		
Total cholesterol (mg/dL)	Borderline high	200–239	1.46	0.41–5.18	0.56
	High	≥240	1.72	0.74–3.98	0.199
Triglycerides (mg/dL)	Low	<150	Ref		
	High	≥150	1.62	0.72–3.68	0.24
HDL cholesterol (mg/dL)	Low	<40	Ref		
	High	≥60	2.66	0.78–9.08	0.78
LDL cholesterol (mg/dL)	Optimal	<100	Ref		
	Near optimal/above optimal	100–129	0.95	0.24–3.82	0.95
	Borderline high	130–159	0.40	0.04–3.57	0.41
	High	160–189	1.12	0.13–10.05	0.92
	Very high	≥190	2.08	0.69–6.33	0.20

**TABLE 8:** The association between disease and colorectal cancer.

Variable	OR	95% CI	P value
Hypertension	0.77	0.29–2.05	0.61
Heart disease	1.84	0.69–4.91	0.22
Stroke	1.71	0.23–12.68	0.60
Diabetes	0.69	0.09–5.07	0.71
Glucose≥126.0 mg/dL	1.40	0.64–3.06	0.41
HbA1c≥6.5%	1.82	0.83–3.99	0.13
Arthritis	0.62	0.28–1.37	0.24
Asthma	0.90	0.12–6.68	0.92
hsCRP (mg/L), mean (SD)	1.00	0.96–1.06	0.72
Chronic lung disease	2.79	1.11–6.99	0.03
Stomach disease	1.96	0.87–4.46	0.10
Liver disease	2.15	0.51–9.16	0.30

### Multivariate logistic regression

We selected the population’s baseline age, gender, BMI, smoking, drinking, night sleeping hours, and disease status: heart disease, hyperlipidemia, arthritis, chronic lung disease, and liver disease for multiple regression analysis. No significant factors were found concerning the prevalence of colorectal cancer. The modelling results are shown in Table 9.

**TABLE 9:** The result of multivariate logistic regression.

Variables	Category	OR	95% CI		P value
Baseline age	<=59	Ref			
	60–69	0.82	0.30	2.20	0.69
	>=70	1.39	0.50	3.89	0.53
Sex	Female	Ref			
	Male	1.94	0.63	5.98	0.25
BMI	Normal (18.5–24.9)	Ref			
	Low (<18.5)	1.21	0.27	5.54	0.80
	Pre-obese (25–29.9)	0.61	0.17	2.19	0.44
	Obese (>=30)	1.08	0.42	2.76	0.87
Smoking	No	Ref			
	Yes	1.67	0.58	4.80	0.34
Drinking	No	Ref			
	Yes	0.62	0.25	1.54	0.30
Night Sleeping hours	7–9 h	Ref			
	<7 h	0.73	0.33	1.63	0.45
	>9 h	—			
Hypertension	No	Ref			
	Yes	0.64	0.22	1.85	0.41
Heart disease	No	Ref			
	Yes	1.62	0.56	4.70	0.38
Dyslipidemia	No	Ref			
	Yes	2.20	0.69	7.03	0.18
Diabetes	No	Ref			
	Yes	0.58	0.07	4.55	0.60
Arthritis	No	Ref			
	Yes	0.63	0.28	1.43	0.27
Asthma	No				
	Yes	2.02	0.25	16.42	0.51
Chronic lung disease	No	Ref			
	Yes	2.27	0.83	6.19	0.11
Liver disease	No	Ref			
	Yes	1.57	0.36	6.93	0.55

### Cox proportional hazards (Cox) model

Based on the univariate analysis of Cox’s model (Table 10), the Hazard Ratio (HR) for the 60–69 age group, as compared to the reference group of individuals aged ≤59 years, was found to be 0.97. However, statistical analysis did not reveal any significant difference in risk between these two groups (p = 0.96). In the 70-year-old age group, the risk of developing the illness was estimated to be 1.58 times higher than that of the reference group (≤59 years old). Nonetheless, given that the confidence interval includes the value of 1 and the p-value is high, it is not possible to conclude a significant correlation exists between this factor and the occurrence of illness within this dataset. The risk of contracting the illness among smokers was 2.3 times greater than that of non-smokers (p = 0.04). Individuals with chronic lung disease displayed a risk approximately 2.79 times higher than those without lung disease (p = 0.03). No significant correlations were observed between other factors examined and disease risk. The Cox multivariate regression analysis findings aligned closely with those obtained through multiple linear regression, ultimately identifying no variables that demonstrated statistical significance. Multiple analyses of the Cox model are shown in Supplementary Table 1.

**TABLE 10:** Univariate analysis of Cox model

Variables	Category	HR	95% CI		P value
Baseline age	<=59	Ref			
	60–69	0.97	0.37	2.56	0.96
	>=70	1.58	0.60	4.15	0.36
Sex	Female	Ref			
	Male	2.31	1.00	5.36	0.05
BMI	Normal (18.5–24.9)	Ref			
	Low (<18.5)	1.36	0.31	6.02	0.69
	Pre-obese (25–29.9)	0.55	0.16	1.94	0.36
	Obese (>=30)	0.95	0.38	2.38	0.92
Smoking	No	Ref			
	Yes	2.30	1.03	5.13	0.04
Drinking	No	Ref			
	Yes	0.96	0.42	2.23	0.93
Night Sleeping hours	7–9 h	ref			
	<7 h	0.82	0.37	1.79	0.62
	>9 h	—			
Hypertension	No	ref			
	Yes	0.77	0.29	2.05	0.61
Heart disease	No	Ref			
	Yes	1.84	0.69	4.90	0.22
Dyslipidemia	No	Ref			
	Yes	1.86	0.64	5.43	0.25
Diabetes	No	Ref			
	Yes	0.69	0.09	5.07	0.71
Arthritis	No	Ref			
	Yes	0.62	0.28	1.37	0.24
Asthma	No	Reg			
	Yes	0.90	0.12	6.67	0.92
Chronic lung disease	No	Ref			
	Yes	2.79	1.11	6.97	0.03
Liver disease	No	Ref			
	Yes	2.16	0.51	9.15	0.29

## Discussion

The incidence and mortality rate of colorectal cancer is increasing year by year globally (Hossain et al., 2022; Xi and Xu, 2021). The incidence rate of colorectal cancer in the Chinese population is significantly increasing (Qu et al., 2022). The high-fat and low fiber diet, lack of exercise, and family history of colorectal cancer are the reasons for the high incidence of colorectal cancer (Li et al., 2022). In order to accurately understand the factors that affect the incidence of colorectal cancer and take corresponding measures, physiological indicators such as gender, age, height, weight, waist circumference, waist-to-height ratio, and BMI; The relationship between smoking, drinking, exercise, sleep, self-reported lifestyle indicators, hypertension, cardiovascular disease, dyslipidemia, diabetes, immune system diseases, chronic lung disease, stomach disease, liver disease and colorectal cancer incidence rate was statistically analyzed from the CHARLS database. The CHARLS cohort is nationally representative of the Chinese population aged 45 and above, providing robust data for studying colorectal cancer (CRC) risk factors in this demographic. However, the generalizability of our findings to populations outside China may be limited due to differences in genetic predispositions, lifestyle factors, and environmental exposures. For instance, dietary habits and the prevalence of certain risk factors may vary significantly between regions. Additionally, the CHARLS cohort primarily includes middle-aged and older adults, which may limit the applicability of our findings to younger populations.

The univariate regression analysis revealed that smoking, chronic lung disease and age to quit drinking were significant risk factors for colorectal cancer. However, no significant factors were associated with colorectal cancer risk when considering multiple factors simultaneously through a multiple regression model. The lack of significance in the multivariate analysis suggests that confounding variables may have influenced the observed associations in the univariate analysis. In the Cox regression model, the univariate regression analysis revealed a statistically significant association between smoking, chronic lung disease, and the incidence of colorectal cancer. However, upon conducting the multivariate regression analysis, no variables were identified as significantly associated with colorectal cancer. However, this study’s results are inconsistent with the conclusions found in many studies. Smoking may increase colorectal cancer risk through various mechanisms: on one hand, numerous carcinogens in tobacco (such as polycyclic aromatic hydrocarbons and nitrosamines) can directly damage the DNA of colorectal epithelial cells, inducing gene mutations; on the other hand, smoking can also impair immune function, weakening the body’s immune surveillance and clearance of cancer cells, thereby promoting the occurrence and development of colorectal cancer. Chronic lung disease patients often experience chronic inflammation, and the large amounts of reactive oxygen species (ROS) and pro-inflammatory factors (such as tumor necrosis factor α and interleukin 6) produced during inflammation can damage colorectal tissue, promote cell proliferation and gene mutations, and increase the risk of colorectal cancer. However, due to the sample size limitations in this study, these factors did not maintain significance in the multivariate analysis. Future studies with larger scales should further explore the relationship between smoking, chronic lung disease, and colorectal cancer, as well as their biological mechanisms.

Studies (Loke and Chuah, 2022; Otani et al., 2005; Stegeman et al., 2013) consistently suggest that older age and higher BMI are associated with an increased risk of colorectal cancer. While there is some indication that being male may confer a slightly higher risk, the evidence remains inconclusive. Previous beliefs regarding the relationships between waist circumference, waist-to-height ratio, BMI, and colorectal cancer risk may be contingent upon differences in study populations. Moreover, these indicators, particularly BMI, may not adequately capture body fat distribution. Variations in fat distribution between genders, with males exhibiting more significant visceral fat deposition and females showing subcutaneous fat accumulation, can impact fat metabolism, cytokine secretion, and hormone levels and consequently influence tumor development (Heo et al., 2021). Smoking and alcohol consumption has also been reported as potential risk factors for colorectal cancer (Akter et al., 2021; Chen H. D. et al., 2019; Huang et al., 2022), although the magnitude of the effect may vary across studies due to differences in study design and measurement of exposure. Multiple researches (Kerr et al., 2017; Kohler et al., 2016; McTiernan et al., 2019; Mehra et al., 2017; Yerrakalva et al., 2023) highlight the association between physical activity and a reduced risk of colorectal cancer and improved survival rates for those already diagnosed with the disease. Regular exercise has consistently shown promise as a protective factor. However, variations in study methods and population characteristics make it challenging to draw definitive conclusions regarding the optimal dose, intensity, and type of exercise for maximum benefit. Some studies (Chen et al., 2022; Lin et al., 2018; Tran et al., 2023; Thompson et al., 2011) have explored the correlation between sleep duration and the prevalence of colorectal cancer. Preliminary findings suggest that short and long sleep duration may be associated with an elevated risk (Lin et al., 2018; Tran et al., 2023). However, the evidence in this area is limited and inconsistent. Further research is necessary to establish causality and understand this association’s underlying mechanisms. Within colorectal cancer research, there is significant interest in studying biomarkers present in venous blood samples (Duijnhoven et al., 2011; Fang et al., 2021; Wang et al., 2017). These biomarkers hold promise for early detection, prognosis, and targeted therapies.

Our study found that BMI was not significantly associated with colorectal cancer, which contradicts numerous other studies. Possible reasons include: firstly, differences in population characteristics across studies, as our study focuses on middle-aged and older Chinese individuals, while other studies may involve different age, gender, or ethnic groups, leading to inconsistencies in the relationship between BMI and colorectal cancer; secondly, BMI as an indicator of obesity may not fully reflect body fat distribution, which could be more critical in influencing colorectal cancer risk; moreover, dietary habits and lifestyle factors may interact with BMI to affect colorectal cancer incidence, and the degree of control for these factors varies across studies, potentially contributing to the differences in results. Future research should further investigate these potential factors and validate the relationship between BMI and colorectal cancer in more diverse populations.

This study has several limitations that should be considered when interpreting the findings. Firstly, using a relatively small database limits the statistical power and generalizability of the results. A larger database would have provided more precise estimates and allowed for subgroup analyses, enhancing the validity of the findings. Secondly, colorectal cancer incidence is comparatively low compared to other chronic diseases such as diabetes and heart disease. This lower incidence may have affected the study’s ability to detect significant associations and necessitates larger sample sizes or longer follow-up periods to capture sufficient cases. Finally, the reliance on risk factor data collected at baseline in 2011 raises concerns about temporal changes in these factors during the observation period. As risk factors can evolve, failing to account for such changes may introduce bias and limit the accuracy of the conclusions. Longitudinal studies with repeated assessments of risk factors would provide more robust insights into the dynamic relationship between these factors and colorectal cancer incidence. Self-reporting bias may have influenced the accuracy of our findings, particularly for variables such as smoking, alcohol consumption, and physical activity. While we employed validation measures, including cross-checking with medical records and using standardized questionnaires, some degree of bias may still be present. Future studies should consider incorporating additional validation measures to further reduce the impact of self-reporting bias. A power analysis was conducted to ensure that our study had sufficient statistical power to detect meaningful associations. Based on previous studies and the expected effect sizes for the primary risk factors, we determined that our sample size of 17,662 participants provided adequate power (80%) to detect significant associations with a two-sided alpha level of 0.05. However, we acknowledge that the number of colorectal cancer cases (n = 25) is relatively small, which may limit the power to detect significant associations in multivariate analyses. Future studies with larger sample sizes are needed to further validate our findings. Our study did not include detailed dietary variables, which are significant for CRC risk. This limitation may affect the comprehensiveness of our analysis. Future analyses will incorporate detailed dietary data to provide a more comprehensive understanding of CRC risk factors.

This study exhibits several notable strengths that contribute to its scientific rigour and reliability. Firstly, the questionnaire design of CHARLS draws on international experience represented by the United States Health and Retirement Survey (HRS), the United Kingdom Elder Tracking Survey (ELSA), and the European Health, Aging, and Retirement Survey (SHARE). It is currently the most comprehensive aging data in China and provides the possibility for international comparative research. Utilizing a national representative longitudinal database CHARLS enhances the study’s external validity and generalizability of findings. Drawing data from a diverse sample representative of the entire population increases the likelihood of capturing a wide range of experiences and risk factors associated with colorectal cancer. Secondly, the extensive observational period from 2011 to 2018 allows for a comprehensive examination of the long-term associations between risk factors and colorectal cancer incidence. This extended timeframe provides a more thorough understanding of the temporal relationships and potential latency periods involved in developing the disease. Lastly, the biomarkers in the data are evaluated by laboratory testing rather than self-reported measures, ensuring the accuracy and reliability of the data. Objective laboratory tests reduce potential biases inherent in self-reporting, providing more robust evidence on the relationship between specific risk factors and colorectal cancer.

## Conclusion

This study provides a comprehensive overview of the current understanding of colorectal cancer risk factors. Through the analysis of middle-aged and older Chinese individuals in this study, we found significant associations between colorectal cancer and factors such as smoking and chronic lung disease in the univariate analysis. Although these associations were not confirmed in the multivariate analysis due to sample size limitations, they provide important clues for potential risk factors of colorectal cancer. Future research should focus on conducting larger-scale cohort studies to validate the relationship between these potential risk factors and colorectal cancer and to explore their biological mechanisms in depth, thereby providing a scientific basis for developing more effective prevention strategies for colorectal cancer.

## Data Availability

Publicly available datasets were analyzed in this study. This data can be found here: https://charls.charlsdata.com/index/zh-cn.html.
