# Supplementary material for: Risk factors of colorectal cancer in middle-aged and elder adults in China: findings from the China health and retirement longitudinal study
Source: Front Mol Biosci. 2025 Apr 25;12:1333834. doi: 10.3389/fmolb.2025.1333834 (PMC12061671; doi:10.3389/fmolb.2025.1333834)
Supplement: Supplementary file 1 [file Table1.docx]

Supplementary Table 1: Multiple analysis of Cox's model

| **Variables** | **Category** | **OR** | **95% CI** | | **P value** |
| --- | --- | --- | --- | --- | --- |
| Baseline age | <=59 | ref |  |  |  |
|  | 60-69 | 0.82 | 0.30 | 2.20 | 0.69 |
|  | >=70 | 1.39 | 0.50 | 3.87 | 0.53 |
| Sex | Female | ref |  |  |  |
|  | Male | 1.93 | 0.63 | 5.97 | 0.25 |
| BMI | Normal (18.5-24.9) | ref |  |  |  |
|  | Low (<18.5) | 1.21 | 0.27 | 5.52 | 0.80 |
|  | Pre-obese (25-29.9) | 0.61 | 0.17 | 2.18 | 0.44 |
|  | Obese (>=30) | 1.08 | 0.42 | 2.76 | 0.87 |
| Smoking | No | ref |  |  |  |
|  | Yes | 1.67 | 0.58 | 4.80 | 0.34 |
| Drinking | No | ref |  |  |  |
|  | Yes | 0.62 | 0.25 | 1.54 | 0.30 |
| Night Sleeping hours | 7-9 hours | ref |  |  |  |
|  | <7 hours | 0.73 | 0.33 | 1.63 | 0.46 |
|  | >9 hours | - |  |  |  |
| Hypertension | No | ref |  |  |  |
|  | Yes | 0.64 | 0.22 | 1.85 | 0.41 |
| Heart disease | No | ref |  |  |  |
|  | Yes | 1.62 | 0.56 | 4.70 | 0.38 |
| Dyslipidemia | No | ref |  |  |  |
|  | Yes | 2.20 | 0.69 | 6.99 | 0.18 |
| Diabetes | No | ref |  |  |  |
|  | Yes | 0.58 | 0.07 | 4.54 | 0.60 |
| Arthritis | No | ref |  |  |  |
|  | Yes | 0.63 | 0.28 | 1.43 | 0.27 |
| Asthma | No |  |  |  |  |
|  | Yes | 2.02 | 0.25 | 16.30 | 0.51 |
| Chronic lung disease | No | ref |  |  |  |
|  | Yes | 2.27 | 0.83 | 6.15 | 0.11 |
| Liver disease | No | ref |  |  |  |
|  | Yes | 1.57 | 0.36 | 6.89 | 0.55 |
